# Viral hijacking of cellular metabolism

**DOI:** 10.1186/s12915-019-0678-9

**Published:** 2019-07-18

**Authors:** Shivani K. Thaker, James Ch’ng, Heather R. Christofk

**Affiliations:** 1 Department of Biological Chemistry, David Geffen School of Medicine, University of California, Los Angeles (UCLA), Los Angeles, CA 90095 USA; 2 Department of Pediatrics, Division of Hematology/Oncology, David Geffen School of Medicine, UCLA, Los Angeles, CA 90095 USA; 3 Jonsson Comprehensive Cancer Center, UCLA, Los Angeles, CA 90095 USA; 4 Eli and Edythe Broad Center of Regenerative Medicine and Stem Cell Research, UCLA, Los Angeles, CA 90095 USA

## Abstract

This review discusses the current state of the viral metabolism field and gaps in knowledge that will be important for future studies to investigate. We discuss metabolic rewiring caused by viruses, the influence of oncogenic viruses on host cell metabolism, and the use of viruses as guides to identify critical metabolic nodes for cancer anabolism. We also discuss the need for more mechanistic studies identifying viral proteins responsible for metabolic hijacking and for in vivo studies of viral-induced metabolic rewiring. Improved technologies for detailed metabolic measurements and genetic manipulation will lead to important discoveries over the next decade.

## Introduction

**Fig. 1:**
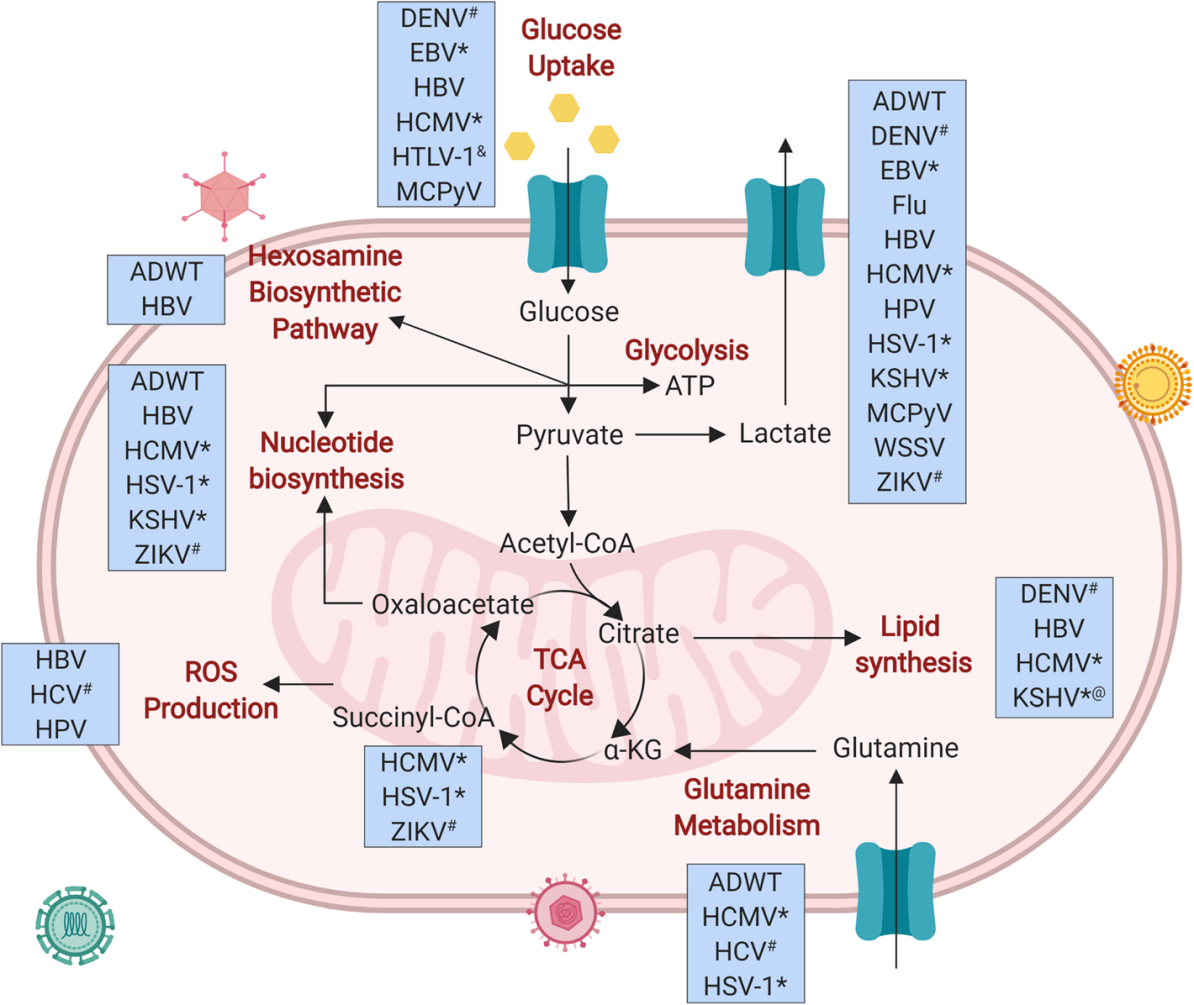
Metabolic pathways altered by virus infection. Figure includes alterations demonstrated by changes in metabolite levels, flux, and tracing. *Herpesvirus family; ^#^Flavivirus family; ^&^virus downregulates this metabolic activity; ^@^KSHV upregulates lipid synthesis but downregulates cholesterol synthesis. Created with BioRender.com

Although it’s been known for over half a century that viral infection alters host cell metabolism, the mechanisms and consequences of virus-induced metabolic reprogramming have only begun to be studied in detail over the past decade (Fig. 1). Viruses clearly rely on host cell machinery to propagate—they promote anabolism for generation of macromolecules needed for virion replication and assembly. Therefore, it is not surprising that viral infection triggers metabolic reprogramming in host cells to facilitate optimal virus production. Metabolic phenotypes conferred by virus infection often mirror metabolic changes seen in cancer cells, such as upregulation of nutrient consumption and anabolism to support viral replication or rapid cell growth, respectively. For example, cancer cells and virus-infected cells commonly both exhibit the Warburg effect: increased glycolytic metabolism in the presence of adequate oxygen for oxidative phosphorylation, to supply reducing equivalents and precursors for macromolecule biosynthesis [1, 2]. Increased nucleotide and lipid biosynthesis are two other metabolic alterations associated with tumorigenesis and rapid cell proliferation that are also seen in various virus infections [1–8]. However, it remains to be determined whether metabolic reprogramming by cancer-causing viruses contributes to oncogenesis. Here we discuss what is currently known about the metabolic reprogramming by different viruses, the effects of oncogenic viruses on host cell metabolism, and the use of viruses as a guide to identify critical metabolic nodes for cancer anabolism. Throughout, we point out gaps in knowledge and important unknowns in the viral metabolism field that will hopefully be elucidated in future studies.

## Virus infection induces metabolic reprogramming in host cells

**Fig. 2:**
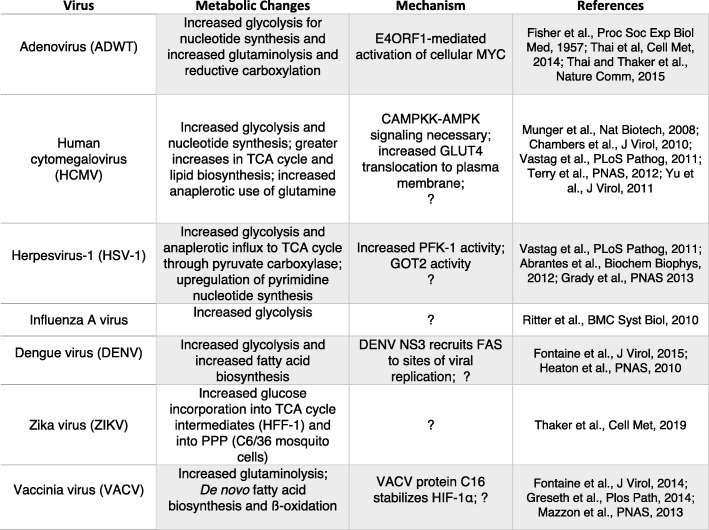
Non-oncogenic viruses and metabolic alterations in host cells during infection

In this section, we describe what is currently known about how different viruses rewire host cell metabolism to facilitate optimal viral replication. Both DNA and RNA viruses have been shown to reprogram various aspects of host central carbon metabolism, including increased glycolysis, elevated pentose phosphate activity to support generation of nucleotides, amino acid generation, and lipid synthesis (Fig. 2). While several viruses upregulate consumption of key nutrients like glucose and glutamine and converge on similar metabolic pathways for anabolism, the precise metabolic changes induced by specific viruses are often context-dependent and can vary even within the same family of viruses or depend on the host cell type that is infected. While improved technologies have enabled a more in-depth analysis of how different viruses alter host cell metabolism to promote virus replication, future studies are needed to further uncover mechanisms involved in viral metabolic reprogramming.

### Adenovirus

Adenovirus is a double-stranded DNA virus that relies entirely on host cell machinery for replication [9]. Several early studies in the 1950s through 1970s described increases in glycolysis during adenovirus infection [10, 11]. However, recent technological advances have enabled more detailed analysis of the metabolic changes induced during adenovirus infection, and potential mechanisms by which metabolic reprogramming may occur. Wild-type adenovirus 5 (ADWT) infection of human breast and bronchial epithelial cells leads to increased glucose consumption and lactate production as well as decreased oxygen consumption rates [2]. Glucose is used to generate pentose phosphate pathway intermediates and nucleotides during infection, likely to support viral genome replication [2]. The ADWT-induced increases in glycolysis are mediated by early adenovirus gene product E4ORF1 binding to cellular MYC to direct transcription of specific glycolytic enzymes, including HK2 and PFKM, and an adenovirus containing the D68A point mutation in E4ORF1 that prevents binding to MYC does not replicate as well as ADWT [2].

In addition to altering cellular glucose metabolism, ADWT infection of human bronchial epithelial cells results in increased glutamine consumption and activity of glutaminase (GLS) [12]. Glutamine tracing studies show that glutamine undergoes reductive carboxylation during ADWT infection, potentially as a source of citrate [12]. Additionally, glutamine is used to generate amino acids and hexosamine pathway intermediates [12]. These changes in glutamine metabolism are all dependent on E4ORF1 binding to cellular MYC. Pharmacologic inhibition of GLS by CB-839 reduces optimal replication of not only adenovirus, but also diverse viruses including HSV-1 and influenza A virus [12].

Although adenovirus-encoded E4ORF1 activation of MYC is responsible for the reported changes in glucose and glutamine metabolism during viral infection, the reduced respiration in adenovirus-infected cells seems to occur independent of E4ORF1 and MYC. The D68A mutant adenovirus deficient in E4ORF1 binding to MYC reduces respiration in infected human breast epithelial cells [2]. It will be interesting for future studies to identify the molecular mechanism by which adenovirus alters host cell respiration and to decipher whether and how this may be beneficial for viral replication.

### Herpes family (HSV-1, HCMV)

Herpesviruses are DNA viruses that undergo both lytic and latent phases of their viral reproduction cycle. While there are more than 100 known herpesviruses, about eight are known to infect human cells exclusively, and can lead to latent infection in specific human tissues [13].

#### Herpes simplex virus 1 and 2

Herpes simplex virus 1 and 2 (HSV-1 and HSV-2) are common viruses that typically cause cold sores and genital herpes, respectively. After entry into the host, both viruses replicate in epithelial cells before ascending into the neural ganglia where latent infection is established [13]. More is known about the metabolic reprogramming conferred by HSV-1 infection than HSV-2 infection.

HSV-1 alters glucose metabolism variably in different contexts. A study in the 1960s showed that HSV-1 virus production is impaired in the absence of glucose in growth media [14]. HSV-1 infection of human foreskin fibroblast (HFF) cells by the KOS strain of HSV-1 does not lead to a change in glucose consumption and lactate production [3]. However, infection of African monkey kidney epithelial (Vero) cells by the acyclovir-resistant AR-29 HSV-1 strain promotes increased glucose consumption and lactate production as well as increased phosphofructokinase (PFK-1) activity and expression [15]. The variable metabolic phenotypes may partially be context-dependent since different cell types and HSV-1 viral strains were used in both studies.

Recent studies utilizing liquid chromatography coupled to mass spectrometry (LC-MS) and isotope tracers have analyzed the metabolic changes induced by HSV-1 infection of host cells. HSV-1 infection leads to increased levels of glycolytic intermediates upstream of phosphoenolpyruvate (PEP) [3]. Notable metabolic phenotypes of HSV-1 infected cells include increased levels of pentose phosphate pathway intermediates and deoxypyrimidines and increased kinetic labeling of UTP by U-^13^C_5_-glutamine, indicative of elevated de novo pyrimidine nucleotide synthesis [3]. HSV-1 upregulates pyrimidine nucleotide biosynthesis by increasing aspartate generation, both through elevated glucose flux into the TCA cycle through pyruvate carboxylase (PC) and from glutamine anaplerosis, followed by subsequent metabolism by glutamic-oxaloacetic acid transaminase 2 (GOT2) to form aspartate, which contributes to the pyrimidine backbone [3, 16]. Consistently, knockdown of PC and GOT2 decrease optimal HSV-1 replication [3]. A long noncoding RNA, lncRNA-ACOD1, binds GOT2 and enhances its catalytic activity while deficiency of the lncRNA decreases viral replication of HSV-1, vaccinia virus, and vesicular stomatitis virus [17]. Since aspartate is an important source of carbons for nucleotide synthesis, knockdown of enzymes that deplete aspartate, including argininosuccinate synthetase (AS1), increases viral titers by increasing aspartate availability for virus replication [16].

Since HSV-1 is a large double-stranded DNA virus with a genome of approximately 152 base pairs, viral replication necessitates a large pool of nucleotides [18]. Interestingly, HSV-1 encodes some of its own nucleotide metabolism enzymes, including thymidine kinase, dUTPase, uracil-DNA glycosylase, and ribonucleotide reductase [19]. It has been hypothesized that HSV-1 has evolved to promote nucleotide biosynthesis in host cells since the virus infects and replicates in non-proliferative cell types such as neurons. Consistent with evidence of nucleotide metabolism playing an important role in HSV-1 replication, current first-line therapeutics to treat HSV infections include nucleoside analogs such as acyclovir and ganciclovir, which exert their effects on infected cells after being metabolized by viral thymidine kinase [20]. However, resistance to these conventional drugs has been emerging, and a better understanding of whether and how drug-resistant HSV strains reprogram host cell metabolism, including AR-29, a strain of HSV-1 that is resistant to acyclovir, could lend insight to treating resistant infections [21].

Whether modulation of glycolysis impacts HSV-1 replication is not completely understood. Treatment of HSV-infected human embryonic lung cells with 2-deoxyglucose (2-DG), a commonly used inhibitor of glucose metabolism, leads to reduced glycosylation of viral glycoproteins and decreased viral-induced cell fusion, which is important for cell-to-cell spread of HSV [22]. 2-DG treatment has also been shown to attenuate HSV-1 replication [23, 24], and a clinical trial in which female patients with genital herpes were treated with either placebo drug or 2-DG for a 3-week period concluded that 2-DG decreases viral shedding and improves symptoms [25]. However, this finding was directly challenged by another group that argued that 2-DG treatment is not efficacious in reducing HSV-1 and HSV-2 virulence in mouse and guinea pig models [26]. Inhibition of glycolysis through genetic means may help shed light on whether or not glucose metabolism is important for HSV replication. Future studies examining metabolic effects of different strains of HSV-1 on host cells as well as tropism based on cell type would enhance understanding of HSV-induced metabolic reprogramming.

#### Human cytomegalovirus

Infection by human cytomegalovirus (HCMV), another herpesvirus family member, is asymptomatic in immunocompetent adults but can lead to more serious complications like retinitis, interstitial pneumonitis, and encephalitis in immunocompromised patients [27].

HCMV promotes increased glucose consumption and lactate production in infected human fibroblasts, and glucose withdrawal during infection reduces virus replication [3, 28–31]. HCMV promotes increased glycolytic flux [29], likely through inducing increased expression of several glycolytic enzymes and activity of PFK [32]. Mechanistically, HCMV-encoded major immediate-early protein IE72 alters expression of glucose transporters in infected cells—it eliminates GLUT1 protein and, by an unknown mechanism, increases mRNA and protein levels of GLUT4, which has three times higher affinity for glucose than GLUT1 [30]. Treatment of cells with the drug indinavir, thought to selectively inhibit GLUT4 activity, reduces glucose uptake and HCMV virus replication [30]. GLUT4 upregulation is dependent on carbohydrate-response element binding protein (ChREBP), which is highly elevated at both the mRNA and protein levels during HCMV infection, and ChREBP knockdown reduces GLUT4 mRNA levels and subsequently glucose consumption and lactate production [33]. ChREBP knockdown in host cells also decreases HCMV replication [33]. AMP-activated protein kinase (AMPK) is also more active in HCMV-infected cells, and blocking AMPK signaling reduces glycolysis induced by infection and virus replication [34].

In addition to promoting increased glucose uptake and glycolysis, HCMV also promotes increased U-^13^C_6_-glucose labeling of TCA cycle intermediates, particularly citrate, which is kinetically labeled by glucose more rapidly in HCMV-infected cells compared to mock controls [29]. Studies with U-^13^C_6_-glucose have shown that an increased fraction of glucose is used to generate fatty acid and glycerol moieties of lipids in HCMV-infected cells [3]. Glucose can be used to form acetyl CoA, which is a critical precursor for lipid synthesis and can be generated by ATP-citrate lyase (ACLY) and acetyl-CoA synthetase short-chain family member 2 (ACSS2) [35]. ACSS2, but not ACLY, is important for HCMV-induced lipogenesis and virus replication [35]. HCMV also induces lipogenesis and expression of key lipogenic enzymes during infection through sterol regulatory element binding protein 1 and 2 (SREBP1 and 2) cleavage and activation and induction of PKR-like endoplasmic reticulum (ER) kinase (PERK) [36, 37].

HCMV infection promotes glutaminolysis in infected cells [31]. Consistently, the enzyme activities of glutaminase (GLS) and glutamate dehydrogenase (GDH) are also elevated upon HCMV infection [31]. Since glucose is routed towards lipid synthesis during HCMV infection, glutamine is used to replenish the TCA cycle in host cells and contributes to increased ATP production [31]. Glutamine withdrawal decreases virus titer and ATP production, both of which can be rescued, at least in part, by addition of alpha-ketoglutarate, oxaloacetate, or pyruvate [31].

### Flaviviruses

Flaviviruses are a genus of positive, single-stranded RNA viruses that are typically transmitted to humans through arthropod vectors, including mosquitoes, and undergo lytic replication [38]. The *Flaviviridae* family consists of viruses known to cause serious diseases, including dengue virus (DENV), West Nile virus (WNV), Japanese encephalitis virus (JENV), Zika virus (ZIKV), and hepatitis C virus.

#### Dengue virus

As with many other viruses, DENV infection of primary HFF cells increases glycolysis and alters levels of glycolytic intermediates compared to uninfected cells [39]. DENV infection of HFFs also induces upregulation of GLUT1 protein levels and increases in hexokinase 2 (HK2) mRNA and protein levels [39]. Consistently, glucose withdrawal in host HFF cells leads to a nearly 2-log decrease in DENV replication, and 2-DG treatment of HFF and immortalized endothelial (TIME) cells during infection reduces virus replication [39]. Intracellular glutamine and glutamate levels also increase during DENV infection, although glutamine withdrawal in cell medium during infection leads to a minimal change in infectious DENV production [39].

While studies examining glucose utilization during DENV infection via glucose tracers remain to be completed, a likely use of glucose is in the TCA cycle and for citrate production to generate lipids. DENV infection leads to elevated fatty acid synthase (FAS) activity in host cells, and radiolabeled acetate and malonyl-CoA incorporation into lipids increases in infected versus non-infected host human embryonic lung cells [40]. Mechanistically, DENV nonstructural protein 3 (NS3) recruits FAS to sites of DENV particle replication and stimulates FAS activity. Consistently, FAS inhibitors, cerulenin and C75, reduce DENV replication [40]. DENV also induces autophagy to release free fatty acids and increase cellular β-oxidation during infection via stimulation of AMPK signaling [4, 41]. Other flaviviruses, including WNV and JEV, have also been shown to rely on lipid metabolism for optimal replication [42, 43].

#### Zika virus

ZIKV is a flavivirus that has been emerging as a public health concern. It can infect a broad range of cell types, including neural progenitor cells, which may lead to microcephaly and developmental abnormalities in infected fetuses [44].

Metabolically, ZIKV infection of both human and mosquito cells leads to increased glycolysis [45]. ZIKV-infected HFFs use increased glucose to generate TCA cycle intermediates, whereas infected mosquito cells use increased glucose for pentose phosphate pathway generation [45]. Interestingly, nucleotide triphosphates are depleted in human cells but not mosquito cells, leading to AMPK activation and caspase-mediated cell death in human cells [45]. The molecular mechanism by which ZIKV alters specific metabolic pathways in human versus mosquito cells remains to be determined.

### Vaccinia virus

Vaccinia virus (VACV) is a large, enveloped DNA virus and member of the poxvirus family, which includes the variola virus that causes smallpox. VACV is unique in that it replicates in the cytoplasm of the host cell instead of the nucleus like most DNA viruses [46]. One study suggested that VACV infection of HFFs increases intracellular glutamine and glutamate levels at multiple time points post-infection [47]. Consistently, VACV infection of glutamine-starved cells leads to significant decreases in virus replication and treatment of VACV-infected cells with BPTES, a glutaminase inhibitor, also results in reduced virus titers [47]. Exogenous supplementation of α-ketoglutarate, oxaloacetate, or pyruvate during VACV infection in glutamine-depleted media can partially rescue the defects in virus replication, suggesting that glutamine is an important anaplerotic substrate for the TCA cycle during VACV infection. Glutamine withdrawal has no effect on VACV transcription but significantly reduces early and late VACV protein synthesis. Interestingly, glutamine, but not glucose, is a critical nutrient for VACV replication as glucose withdrawal in VACV-infected cells causes no change in viral yield [47]. Future studies using heavy carbon- and nitrogen-labeled glutamine tracers would be interesting to further assess the role of glutamine in VACV-infected cells.

De novo fatty acid (FA) biosynthesis also plays a role in VACV replication. Inhibition of fatty acid synthase by C75 and of acetyl-CoA carboxylase (ACC) by TOFA in VACV-infected cells reduces viral yield, both of which can be partially rescued by exogenous palmitate, the first fatty acid generated from FA synthesis and a precursor to longer fatty acids [48]. Mitochondrial import and β-oxidation of palmitate is important for VACV replication since treatment of infected cells with etomoxir, an inhibitor of mitochondrial import of long-chain fatty acids, and trimetazidine, an inhibitor of β-oxidation, both reduce viral yield compared to DMSO-treated cells. Consistent with VACV-induced β-oxidation, infection of HFFs also leads to increased oxygen consumption and ATP production. Inhibition of mitochondrial import and β-oxidation mildly reduce viral DNA replication and protein synthesis; however, VACV-infected BSC40 cells treated with TOFA and C75 showed decreased viral assembly, suggesting that de novo fatty acid biosynthesis plays an important role in virion assembly.

While VACV has been shown to induce and rely on altered metabolism in host cells, the mechanisms by which VACV causes these changes are still being studied. The VACV genome encodes approximately 200 proteins involved in various processes that assist virus replication. One study showed that the VACV-encoded C16 protein promotes HIF-1α stabilization through binding to the prolylhydroxylase domain-containing protein (PHD)2, a cellular oxygen sensor [49]. Since HIF-1α is an important cellular transcription factor for numerous metabolic genes dysregulated in cancers [50], future studies should explore whether HIF-1α is responsible for the metabolic changes induced during VACV infection.

### Influenza

Influenza A, which is an RNA virus in the *Orthomyxovirus* family, causes acute respiratory disease and is a major public health burden [51].

Influenza A virus has been reported to increase glycolysis, enhancing glucose uptake and lactate production at early time points post-infection, as well as increase oxygen consumption rates [52–54]. Compared to mock-infection, influenza A infection leads to elevated levels of upper glycolytic intermediates and reduced levels of nucleotide triphosphates at early time points [52]. Pediatric patients with respiratory infections showed higher PET signal in influenza A-infected lungs compared to lungs that had cleared the infection and tested influenza negative [54]. Treatment with a putative PI3K/mTOR inhibitor, BEZ235, decreases glycolysis and reduces virus replication at an uncharacterized step following viral genome replication, resulting in decreased mortality in a mouse model of influenza infection [54].

### Miscellaneous viruses

While all the previously mentioned viruses alter the metabolism of host mammalian cells, viruses that infect invertebrate hosts have also been shown to alter metabolism. The invertebrate virus white spot syndrome virus (WSSV) that infects shrimp hemocytes induces glycolysis in infected cells versus normal cells in a PI3K-Akt-mTOR-dependent manner [55, 56]. DENV infection of mosquito cells leads to increased lipid biosynthesis in infected cells [5]. The fact that metabolic reprogramming by viruses is conserved throughout species, even in invertebrates, provides further evidence that metabolic reprogramming is critical for replication of diverse viruses.

### Limitations of current studies and future directions

While diverse viruses have been shown to reprogram host cell metabolism, many of the molecular mechanisms by which viruses induce these changes remain unknown. A number of viruses, in addition to HSV-1, encode their own metabolic enzymes—usually involved in nucleotide biosynthesis—to facilitate viral replication in host cells. For instance, VACV encodes its own thymidylate kinase, and HSV-1 encodes RRM2. Side-by-side comparisons of the virally encoded versus human cell encoded versions of these enzymes may help decipher potential differences in activity or regulation. For those viruses that rely on hijacking host cell machinery for metabolic reprogramming, identifying the viral gene products that interact with host cell factors to modulate metabolism will deepen our understanding of viral-induced changes to host metabolism and may even shed light on viral tropism.

A major limitation in our current understanding of viral-induced metabolic reprogramming stems from the fact that most of the work characterizing viral alterations to host cell metabolism so far has been carried out in vitro. However, metabolism in vivo is known to be quite different from that found in vitro in cell culture conditions. In vivo models to assess metabolic changes induced by virus infection are needed to have a more accurate understanding of viral metabolism and facilitate therapeutic antiviral strategies.

Additionally, many viruses, including HSV-1 and HCMV, undergo both lytic and latent phases of infection in host cells, but most studies characterizing metabolism by both viruses have focused only on the lytic phase. Determining whether metabolic changes are unique at different phases of infection would be of interest.

The interplay between metabolism and the immune system during virus infection is also understudied. Since immune cells also rely on some similar nutrients and pathways perturbed by viruses, understanding the effects of modulating metabolic pathways in vivo will be important in assessing the overall impact to the host. While glutamine metabolism has been shown to be important for HSV-1 replication in vitro [31], glutamine supplementation in HSV-infected mice actually represses reactivation of HSV-1 since it enhances the activity of IFN-γ-producing CD8 T cells [57]. Whether or not an individual’s diet may also impact virus replication would be an interesting area for further investigation.

Future studies should determine whether viruses display tropism for specific cell types and cause different cellular outcomes depending on the metabolic environment and machinery present in the cells. Hints of this concept already exist. For example, HSV-1 increases de novo nucleotide biosynthesis and encodes its own nucleotide metabolism enzymes because HSV-1 infects neurons, which do not actively divide [3]. On the other hand, HCMV typically infects growth-arrested cells, like fibroblasts, and pushes arrested cells into the G1/S cell cycle phase to promote nucleotide synthesis without host cell DNA replication [3]. Additionally, it is intriguing that infection by the same virus can have contrasting effects on different types of host cells, as is the case in ZIKV-infected human versus mosquito cells.

## Influence of oncogenic viruses on host cell metabolism

**Fig. 3:**
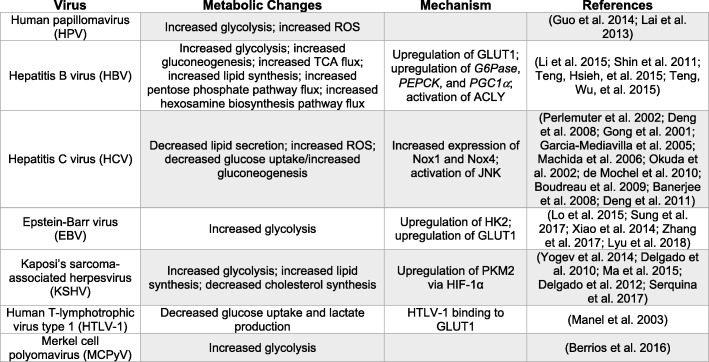
Metabolic reprogramming by oncogenic viruses

Nearly 10% of all new cancer cases worldwide are attributable to oncogenic viruses. These viruses include human papillomavirus (HPV), hepatitis B virus (HBV), hepatitis C virus (HCV), Epstein-Barr virus (EBV), Kaposi’s sarcoma-associated herpesvirus (KSHV), and human T-cell lymphotropic virus type 1 (HTLV-1) (Fig. 3) [58]. Another recently discovered oncogenic virus, Merkel cell polyomavirus (MCPyV), was discovered in 2008 in association with the rare malignancy Merkel cell carcinoma (MCC) [59]. Still other viruses, such as adenovirus type 12, that are not known to be oncogenic in humans have been shown to be capable of inducing transformation and oncogenesis in other animals [60].

As our understanding of how viruses contribute to the development of cancer grows, an emerging area of study is how oncogenic viruses manipulate the metabolism of the host cell. The similarities between the metabolic reprogramming that occurs during viral infection and that which occurs in cancer cells makes the contribution of viral infection to cancer metabolism an important topic. In this section, we discuss the metabolic changes that occur during infection by known oncogenic viruses.

### Human papillomavirus

HPV is a double-stranded DNA virus and is the oncogenic virus found in the largest number of cancer cases, estimated to cause 4.5% of all cancers worldwide, with cervical cancer comprising 83% of these cases while other anogenital cancers and head and neck cancers make up the rest [61].

Studies suggest that HPV infection produces a number of viral proteins that affect host cell metabolism. The HPV viral proteins E6 and E7 augment HIF-1α, which may result in an enhanced glycolytic phenotype in a hypoxic solid tumor microenvironment. E6 stabilizes HIF-1α under hypoxic conditions by inhibiting VHL association with and ubiquitination of HIF-1α [62]. In cells treated with hypoxia mimetic deferoxamine mesylate, E7 is able to enhance HIF-1α activation of target genes [63]. HPV type 16 E7 interacts directly with PKM2 and promotes its dimeric state. This decreases PKM2’s affinity for PEP in the final step of glycolysis and may be a means of diverting glycolytic intermediates for anabolic purposes while compensating for the decreased energy production with upregulated glutamine metabolism [64, 65]. However, this phenomenon has yet to be examined using modern techniques such as metabolite tracing that could confirm this altered nutrient utilization. Additionally, the HPV viral protein E2 has been shown to interact directly with the mitochondrial membrane and induce release of ROS as well as to upregulate HIF-1α [66].

### Hepatitis B virus

HBV is a double-strand DNA virus associated with the development of hepatocellular carcinoma (HCC). The worldwide prevalence of hepatitis B infection is estimated at 257 million people, with the virus and its complications causing 887,000 deaths in 2015 [67]. In 2012, 420,000 new cancer cases were attributed to HBV [58].

HBV infection has broad effects on host cell metabolism, impacting lipid, glucose, amino acid, nucleic acid, vitamin, and bile acid metabolism [68, 69]. A combined metabolomics and proteomics approach to evaluating changes in HBV infection demonstrated evidence for upregulation of hexosamine biosynthesis, phosphatidylcholine biosynthesis, central carbon metabolism, nucleotide synthesis, and oxidative stress [6]. HBV core protein (HBc) has been shown to upregulate multiple metabolic pathways, including glycolysis and amino acid metabolism [70]. Studies have suggested potential specific metabolic effects of HBV viral proteins on glucose metabolism. The HBV pre-S2 mutant protein upregulates GLUT1 expression and plasma membrane localization [71]. HBV X protein (HBx) upregulates G6PD and expression of multiple genes involved in gluconeogenesis [72, 73].

HBV transgenic mice have higher transcription of lipid biosynthesis genes [74]. Similarly, transgenic mice with the HBV pre-S2 mutant antigen exhibited increased lipid droplet accumulation and upregulation of several lipogenic enzymes [7]. HBV X protein (HBx) has been shown to activate lipid synthesis and uptake and inhibit ApoB secretion [75–77].

Other metabolic changes induced by HBV infection include upregulation of proteins involved in the metabolism of retinol, which is interesting because retinoic acid in the presence of retinoic X receptor (RXRα) has been shown to regulate HBV gene expression [78, 79]. HBV infection also enhances proteins involved in the metabolism of cholesterol and biosynthesis of bile acids [80].

### Hepatitis C virus

HCV is a single-stranded RNA virus that is associated with the development of HCC. HCV was estimated to be linked to 170,000 new cases of cancer in 2012 [58]. In contrast to the global predominance of HBV as an etiology of HCC, in some areas, including Japan, Italy, France, and Spain, the majority of HCC cases appear to be associated with HCV infection. In the United States, evidence of HCV infection is found in 30–50% of patients with HCC [81, 82].

HCV infection has been shown to alter metabolism at both the cellular and whole organism level. At the cellular level, major metabolic changes include upregulation of lipogenesis and altered lipid utilization, manipulation of glucose uptake and metabolism, and induction of oxidative stress through mitochondrial dysfunction [83]. Many of the HCV-induced alterations in lipid metabolism stem from the HCV core protein. HCV core protein-expressing transgenic mice develop hepatic steatosis at grades correlative to the HCV core protein level, and subsequent liver lesions with histologic similarity to HCC, suggesting that the core protein plays a role in lipid metabolism rewiring that occurs at an organ level preceding HCC development [84, 85]. HCV core protein within the cell accumulates in a globular pattern around the lipid droplets by means of interaction with DGAT1, and DGAT1−/− mice do not develop steatosis induced by HCV core protein [86–88]. Studies have suggested that HCV core protein can alter lipid metabolism through inhibition of microsomal triglyceride transfer protein (MTP), activation of the *Srebp-1c* promoter (HCV nonstructural protein 2 has also been suggested to perform this function), and increasing proteolytic cleavage of sterol regulatory element binding proteins to their mature forms (HCV nonstructural protein S4B has also been suggested to play a role in this) among others [89–92]. Additionally, transcriptomics studies suggest that the HCV microRNA miR-146a-5p upregulates transcription of genes involved in fatty acid metabolism [93].

HCV infection is associated with induction of oxidative stress and altered maintenance of redox balance. In HCV core protein transgenic mouse models, mice were found to have core protein accumulation at the mitochondrial membrane, higher levels of liver ROS, lower ratios of reduced to total glutathione, and increased signs of oxidative damage, including higher levels of lipid peroxidation and earlier signs of mtDNA damage [94, 95]. HCV-infected cells have increased superoxide production [96], and studies suggest that the HCV core protein and HCV nonstructural proteins NS5A and NS3 are capable of inducing increased ROS [97–100]. HCV core protein may induce ROS by inhibiting mitochondrial complex I, which disrupts the electron transport chain and generates ROS [95]. HCV core protein interacts with the mitochondrial chaperone protein prohibitin, increasing its stability and levels but impairing its ability to interact with cytochrome c oxidase (COX) subunits, potentially resulting in disrupted COX assembly that could lead to increased ROS [101]. HCV core protein increases mitochondrial Ca^2+^ influx, which is thought to induce ROS production [95, 102]. HCV infection increases expression of NADPH oxidases Nox1 and Nox4 via TGFβ1, resulting in increased ROS production, and HCV core on its own appears to increase ROS production via TGFβ1-mediated increases in Nox4 expression and activity [103, 104].

HCV infection perturbs glucose metabolism, resulting in increased insulin resistance and gluconeogenesis. This is manifested clinically in patients with HCV infection as those with sustained responses to antiviral therapy demonstrate decreased insulin resistance and increased IRS1/2 expression [105]. Transgenic mice with expression of HCV core protein in the liver demonstrate evidence of increased insulin resistance [106]. At the cellular level, HCV core protein has been observed to increase IRS1 phosphorylation and impair insulin activation of Akt [107]. Core also decreases IRS1 and IRS2 levels and inhibits 6-phosphofructo-2-kinase activation [108]. The HCV nonstructural protein NS5A has been found to increase hepatic gluconeogenesis through induction of ROS, leading to increased PEPCK and G6Pase expression and decreased glucokinase expression [109].

HCV is also capable of altering glutamine metabolism, and recent studies suggest that HCV infection both upregulates enzymes of glutaminolysis and induces glutamine addiction in the infected cell for both cell growth and for HCV viral replication [110].

### Epstein-Barr virus

EBV is an oncogenic γ-herpesvirus associated with multiple malignancies, most prominently lymphoma but also nasopharyngeal carcinoma, gastric carcinoma, and leiomyosarcoma, and an estimated 120,000 new cases of cancer were attributed to EBV infection in 2012 [58, 111].

EBV infection alters host cell glucose metabolism primarily through viral protein LMP1. Studies suggest that LMP1 promotes glycolysis via FGF2 and FGR1 activation and that this mechanism is also important for the infected cells’ transformation characteristics, including proliferation, migration, and invasiveness [112]. LMP1 also enhances glycolysis by upregulating HK2, a change that correlates with increased cell viability and proliferation. Increased HK2 expression was also noted in some cases of EBV-associated NPC and was negatively correlated with survival [113]. LMP1 enhances expression, stability, and plasma localization of GLUT1, contributing to increased glycolysis [114, 115]. Studies also suggest that LMP1 may upregulate glycolysis by repressing HOX genes [116]. LMP1 also promotes glycolysis by upregulating PDK1 and PKM2 via upregulation of HIF-1α [117, 118]. LMP1 promotes HIF-1α stabilization by enhancing the degradation of prolyl HIF-hydroxylases PHD1 and PHD3 [119]. Additionally, the EBV viral proteins EBNA3 and EBNA5 bind to PHD2 and PHD1, respectively, perhaps representing another mechanism by which EBV infection stabilizes HIF-1α to promote glycolysis [120]. EBV infection also produces the miRNA EBV-miR-Bart1-5P that has been shown to promote a glycolytic phenotype [121].

EBV infection alters lipid metabolism in part through EBV-encoded RNAs (EBERs), which leads to upregulation of fatty acid synthase (FAS) and low-density lipoprotein receptor (LDLR) [122]. During lytic reactivation, expression of one of the EBV immediate-early proteins, BRLF1, results in FAS upregulation [123].

Additionally, EBV infection-induced metabolic changes appear to be linked to modulation of the immune response, and studies suggest that LMP1 mediates upregulation of GLUT1 resulting in increased cytokine secretion and expansion of myeloid-derived suppressor cells [114].

### Kaposi’s sarcoma-associated herpesvirus

KSHV, also known as human herpesvirus 8, is an oncogenic γ-herpesvirus known to cause Kaposi’s sarcoma, resulting in 44,000 new cancer cases in 2012, and is also associated with primary effusion lymphoma (PEL) and multicentric Castleman’s disease [58, 124, 125].

KSHV alters host cell glucose metabolism. KSHV infection promotes glycolysis by upregulating HIF-1α and its glycolytic target genes, including *PKM2*, *HK*, *GLUT1*, and *PDK1,* and by reducing mitochondrial biogenesis via targeting of mitochondrial heat shock protein *HSPA9*, and these changes result in increased cell growth [126–128]. Additionally, studies suggest that miRNAs produced during latent KSHV infection are also secreted in exosomes that infiltrate neighboring cells in the microenvironment, inducing glycolysis in these cells and thereby supporting the growth of the latently infected cells [129].

KSHV latent infection induces lipid droplet formation and alters lipid metabolism by upregulating lipid biosynthesis as well as peroxisome biosynthesis and associated proteins involved in very long chain fatty acid metabolism [8, 130, 131]. KSHV viral miRNAs also inhibit cholesterol synthesis, possibly suppressing cellular innate immune functions [132].

Studies suggest that latent KSHV infection also upregulates glutamine metabolism, inducing protein expression of the glutamine transporter SLC1A5 (ASCT2) as well as MondoA and its downstream targets involved in regulation of glutaminolysis and making the infected cells reliant on glutaminolysis for survival [133]. Metabolomics studies have demonstrated increased levels of pentose phosphate pathway intermediates in KSHV-infected cells, suggesting that the viral infection may also alter nucleotide biosynthesis [8].

### Human T-lymphotropic virus type 1

HTLV-1 is an oncogenic virus of the deltaretrovirus family that is estimated to infect approximately 5–10 million people worldwide and is linked to the development of adult T-cell leukemia, estimated to be associated with 3000 new cases of cancer in 2015 [58, 134, 135].

While host cell metabolic factors, particularly the degree of hypoxia, glycolytic function, and electron transport chain function, appear to influence HTLV-1 reactivation and plus-strand transcription, very little is known about how HTLV-1 infection alters cellular metabolism [136]. Studies from over a decade ago suggest that GLUT1 can function as a receptor for HTLV-1 to bind to cells, conferring cell susceptibility to the virus, and that HTLV-1 suppresses glucose consumption and lactate production when binding to GLUT1 [137, 138]. Unfortunately, there have not been many other studies examining the effects of HTLV-1 infection on metabolism, making this an area with great potential for discovery.

### Merkel cell polyomavirus

MCPyV is a relatively recently discovered oncogenic polyomavirus. Despite MCPyV infection being widely prevalent, ranging from 50 to 80% of the population depending on age, its associated malignancy, Merkel cell carcinoma (MCC), is rare [59, 139]. While little is yet known about the metabolism of MCPyV infection, recent studies utilizing transcriptomics have suggested that the MCPyV small tumor antigen (ST) is able to promote a glycolytic phenotype by upregulating multiple glycolytic genes, including *SLC16A1* (MCT1) and *SLC2A1* (GLUT1) [140].

### Limitations of current studies and future directions

In addition to studies describing how viral infections alter host cell metabolism, there have been studies examining how the host cell metabolic environment affects the progression of some oncogenic viral infections, such as EBV. Studies have suggested that in early EBV infection, metabolic stresses such as decreased mitochondrial respiration or autophagic imbalance present a barrier to cell proliferation [141]. Additionally, hypoxia is able to induce EBV lytic reactivation via binding of HIF-1α to the promoter of the EBV lytic reactivation gene *BZLF1* [142]. Hypoxia also induces KSHV lytic replication through the interaction of viral proteins with HIF-1α [143, 144]. Studies suggest that KSHV also relies on different components of host cell metabolic infrastructure for different stages of viral replication, with glycolysis important for viral gene transcription, glutaminolysis important for viral protein translation, and fatty acid synthesis important for virion assembly and release [145]. Upregulation of glycolytic metabolism and lipogenesis appears to be important for maintenance of KSHV latently infected cells as inhibition of these processes results in increased apoptosis in infected cells compared to mock-infected cells [8, 146]. While this review has focused on the effects of viral infections on metabolism, the related question of how existing host cell metabolism affects viral infections is also intriguing as cell metabolic infrastructure can provide receptors for viral access and metabolic sensors can act as transcription factors for viral genes. It will be interesting to determine whether reliance on certain metabolic pathways makes some cells more hospitable for certain viral infections than others.

In some studies, including several referenced in this review, viral proteins are linked to major metabolic regulators without demonstrating that this link is responsible for a metabolic phenotype. For example, in addition to the previously mentioned link to HIF-1α, many studies have shown that EBV infection and its viral proteins are linked to AMPK and MYC [147]. In another example, HBV X protein (HBx) has been shown to activate major metabolic regulators mTORC1 and AMPK [148]. Studies have suggested that despite their well-established connection with metabolism, major metabolic regulators can have shifting levels of metabolic importance between different forms of infection by a single virus such as EBV [149]. This demonstrates the importance of directly connecting suggested mechanisms with metabolic phenotypes to determine which are truly important metabolic pathways during viral infection.

The studies discussed above have laid the groundwork for understanding metabolic alterations by oncogenic viruses. One major question that remains is whether the virally induced metabolic changes, which bear striking similarity to metabolic perturbations in cancer cells, also promote cellular transformation. Proving that these changes are necessary or sufficient for transformation will require well-designed in vivo studies. Careful mechanistic experiments using viral mutants are needed to clarify the metabolic effects of specific viral proteins expressed at physiological levels as well as to truly demonstrate which metabolic changes are important for cell transformation and how they contribute to oncogenesis. Metabolic phenotypes that have been inferred by alterations in metabolic enzymes should be examined using LC-MS and metabolite tracing to strengthen our understanding of nutrient utilization during infection by various viruses.

## Viruses as cancer discovery tools and therapies

DNA viral proteins and tumor cell mutations converge on many of the same molecular pathways to promote viral or cellular replication, including anabolic pathways. Key oncogenes and tumor suppressor genes, including Src kinase, p53, PI3-kinase, and others, were originally discovered by identifying host pathways that DNA viral proteins interact with to promote viral replication [150]. While new tools are continually being developed to further cancer research, cancer genome instability and short evolutionary life-span make it difficult to distinguish passenger versus molecular drivers of tumorigenesis [150]. On the other hand, viruses, such as adenoviruses, have evolved to be efficient and alter key pathways in host cells that enable them to replicate effectively [150].

**Fig. 4:**
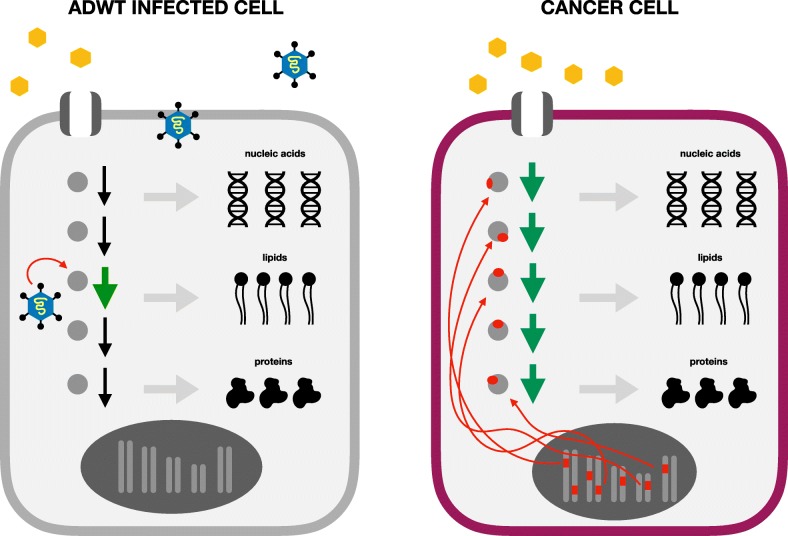
Comparison of a cell infected with wild-type adenovirus and a cancer cell. While cancer cells have numerous mutations and perturbation of whole metabolic pathways (*green*), adenovirus-infected cells upregulate only key metabolic nodes for virion replication

Since adenoviruses have undergone strong selective pressure for efficiency, and they completely rely on host cell machinery for replication, the specific metabolic nodes they hijack for their replicative needs may represent especially important metabolic nodes for anabolism (Fig. 4). As a proof-of-principle, a few years ago we found that adenovirus infection increases GLS protein levels and activity [12]. GLS is already a cancer metabolism drug target—the GLS inhibitor CB-839 is currently being used in clinical trials to treat certain types of solid and hematological malignancies that also rely on GLS activity. We found that treatment of adenovirus infected cells with CB-839 reduces viral titers. Additionally, we also found that adenovirus infection selectively increases expression of asparagine synthetase (ASNS), which is critical for proliferation of various cancer cell lines through its role as an amino acid exchange factor [151]. Adenovirus infection increases ASNS protein expression and knocking down ASNS levels decreases adenovirus replication (unpublished observation). These results are examples that metabolic enzymes and pathways critical for optimal virus replication may also be important for cancer cell proliferation. Understanding how a rapidly replicating virus like adenovirus alters host cell metabolism during infection will likely reveal critical anabolic nodes that are important in different cancers, just as understanding how viruses reprogram host cell signaling and transcriptional machinery helped identify key oncogenes and tumor suppressor genes in the 1970s and 1980s [150].

### Oncolytic viruses

Oncolytic viruses are viruses that are engineered to selectively kill tumor cells and trigger systemic anti-tumor immunity [152]. Compared to traditional approaches to treat cancers, use of oncolytic viruses as a treatment strategy may be advantageous in that normal tissues should not suffer adverse effects to the same extent as with chemotherapeutics due to increased specificity for tumor tissue. Additionally, direct cell lysis by oncolytic viruses would avoid the problem of drug resistance [153]. Despite many potential advantages, however, safety concerns have prevented widespread use of oncolytic viruses as a cancer treatment.

The specificity of tumor cell killing by oncolytic viruses is based on the virus’s requirement for cell functions idiosyncratic to cancer cells, which can include metabolic functions. For instance, adenoviral mutants that are unable to perturb cell cycle machinery, including p53 and retinoblastoma (Rb), needed for replication may replicate in tumor cells that already have dysregulated signaling of both these proteins [150]. Oncolytic virus design could benefit from incorporation of metabolic strategies to achieve better cancer cell specificity. For instance, many DNA viruses have evolved mechanisms to increase dNTP levels in host cells since dNTP levels can be low in cells that are not cycling actively [154]. Oncolytic viruses that lack viral proteins responsible for increased dNTP synthesis or acquisition would be predicted to replicate only in cells that have upregulated dNTP metabolism (i.e., cancer cells). Consistently, the D68A mutant form of adenovirus type 5 deficient in E4ORF1 activation of MYC and increased nucleotide biosynthesis was deficient in replication in primary lung epithelial cells but not in immortalized breast epithelial cells that had enhanced nucleotide biosynthesis rates [2]. A stronger understanding of the mechanisms by which viral proteins perturb metabolism in host cells will be helpful for oncolytic virus design and will improve their selectivity for targeted replication and killing of tumor but not normal cells.

## Conclusions and future directions

While many studies have demonstrated that viruses reprogram cell metabolism and rely on metabolic changes for optimal virus replication in vitro, significant work remains to determine mechanistically what viral proteins interact with host cell machinery to induce such alterations and characterize whether the same metabolic perturbations occur during infection in vivo. Additionally, it will be interesting for future studies to determine whether there is different viral affinity for and replication across tissue types depending on the metabolic environment; whether differential metabolic reprogramming by a virus across multiple species impacts how specific species cope with viral replication; and whether or not viral-induced metabolic reprogramming contributes to oncogenesis. The future is certainly ripe for discovery in the viral metabolism field.

## Data Availability

Not applicable.
